# Novel mercerized *Haloxylon salicornicum* Sahara plants derived adsorbents for efficient removal of lead(II) from wastewater

**DOI:** 10.1038/s41598-025-03791-1

**Published:** 2025-05-31

**Authors:** Magda A. Akl, Asmaa A. Serage, Aya G. Mostafa, Yasser A. El-Amier

**Affiliations:** 1 Department of Chemistry, Faculty of Science, Mansoura University, Mansoura, 35516 Egypt; 2 Botany Department, Faculty of Science, Mansoura University, Mansoura, 35516 Egypt

**Keywords:** Sahara plants-derived adsorbents, *Haloxylon salicornicum*, Adsorption, Pb(II), Error functions, Wastewater treatment, Analytical chemistry, Biosynthesis, Environmental chemistry, Green chemistry, Materials chemistry, Chemical synthesis

## Abstract

In an effort to valorize the use of Egyptian Sahara plants as biomaterials for the heavy metals remediation from water, we present here a thorough investigation of the utilization of the locally accessible *Haloxylon salicornicum* (HS) Sahara plant as an adsorbent for Pb(II) remediation. The raw HS and the mercerized one (HSN) adsorbents were characterized by Boehm’s titration method, Fourier Transform infra-red (FTIR), scanning electron microscopy (SEM), Thermo-gravimetric analysis (TGA), and the pH_PZC_. Many experimental parameters that influence the Pb(II) adsorption onto these adsorbents were thoroughly studied, such as pH, adsorbent (HS and HSN) dose, initial concentration of metal ion, time of contact, and effect of various foreign ions. Due to the higher R^2^ values and the lower values of error functions, the adsorption of Pb(II) onto HS and HSN adsorbents was best described by pseudo-2nd-order and Langmuir isotherm model with adsorption capacities of 100.68 and 113.33 mg/g for HS and HSN, respectively. Thermodynamic studies revealed the spontaneous and endothermic nature of the adsorption of Pb(II). The mechanism of the adsorption of Pb(II) onto HS and HSN adsorbents was elucidated.

**Supplementary Information:**

The online version contains supplementary material available at 10.1038/s41598-025-03791-1.

## Introduction

Recent increases in industrial activities have exacerbated numerous environmental issues, such as the decline of several ecosystems brought on by the buildup of hazardous contaminants such as heavy metals^1^. Apparently, heavy metal pollution has elevated to be one of the most imperative environmental issues of this era as they are stable, cannot be broken down and eliminated, and have the capability of destroying aquatic life. A significant threat to the individual’s health is the presence of harmful metal ions in drinking water. Additionally, heavy metals in streams, lakes, and seas contaminate the water that marine species and humans consume. These pollutants are considered a major risk to human health as they bioaccumulate via the food chain. Arsenic, cadmium, chromium, copper, lead, nickel, and zinc are the main pollutants in water^2^.

The utilization of treated effluents for industrial processes is progressively spreading throughout the world due to the scarcity of freshwater resources. Wastewater reusing after applying a variety of treatment techniques contributes to reducing water shortages, protecting resources for solely potable use, reducing water pollution, and preserving ecosystem life^3,4^.

The determined concentration of discharged Pb(II) in the environment from the battery sector, the mining, and the oil industries is 5–66 mg/L, 0.02–2.50 mg/L, and 125–150 mg/L, respectively^5^. Pb(II) causes lots of illnesses in humans such as anemia, brain damage, mental deficiency, renal damage, encephalopathy, anorexia, cognitive impairment, behavioral difficulties, and vomiting^6,7^. As a consequence of this, it is essential to eliminate Pb(II) from wastewater before discharging it into the environment.

A multitude of wastewater treatment technologies are presently required like coagulation-flocculation^8^, adsorption^9–13^, flotation^14,15^, reverse osmosis^16^, membrane filtration^17^, electrochemical treatment^18^, chemical precipitation^19^, and adsorption^20,21^.

Adsorption is a potential biotechnology that is utilized to remove heavy metals and related organics from waste streams and wastewater^22,23^. Moreover, it valorizes the value of a wide range of agricultural and industrial biomasses by applying them as adsorbents in the process of water treatment^24^. Various biomasses have been utilized as adsorbents for the removal of heavy metal ions like aquatic plant wastes (macro and microalgae), terrestrial plant biomasses (leaves, bark, sawdust), agricultural wastes (fruit peels, cereal straw and shells, etc.), and animal materials (hair, crustaceans)^25–28^. The use of adsorbents provides economic benefits as they are low-cost, readily available in large amounts, and easily renewable^29^.

The adsorption technique has many advantages; it is reversible, effective, fast, independent of the physiological limitations of living cells, does not need innocent conditions, chemical or biological sludge is minimized, and is economically and ecologically similar to traditional technologies that were used for heavy metals remediation from wastewater^30,31^. The plant has the potential to be used as an adsorbent due to the existence of cellulosic functional groups (carboxylic and phenolic groups) in addition to lignin and hemicelluloses (cellulose-linked components).

*H. Salicornicum* (HS), which belongs to the family *Chenopodiaceae*, is widely distributed in North Africa and Asia in temperate and tropical regions with 25 species where it grows in sandy and rocky desert regions as a diffuse shrub^32,33^. *H. Salicornicum, *Fig. S1, is a widespread perennial herb in Egypt. *H. Salicornicum* is known to include several alkaloids such as haloxynine, haloxine, and piperidine alkaloids^34,35^. Additionally, *H. Salicornicum* contains tannins, saponins, various glycosides^36^, and pyranone^37^.

The mercerization process, alkaline treatment, is regarded as one of the most well-known chemical treatment techniques that are applied to plant fibers. This process involves the immersion of plant fibers in NaOH solution. Mercerization is able to modify the surface of fibers through the removal of a certain amount of less dense non-cellulosic components, such as lignin, hemicellulose, wax, and oils that cover the external surface of the plant fibers. The removal of these less dense components leads to an increase in the density of the mercerized fibers. In addition to the breaking of H-bonds and weak Van der Waal forces between the chains of the cellulose, these chains become more arranged after the mercerization^38^.

This investigation aims to evaluate the potential use of raw (HS) and NaOH modified(HSN) forms of *H. Salicornicum* as eco-friendly and cheap adsorbents for removing Pb(II) from aqueous solutions under optimum conditions.

To the best of our knowledge, the mercerization of *H. Salicornicum* (HS) utilizing NaOH has not been reported in the literature. Furthermore, the HS and HSN adsorbents are utilized for the first time as adsorbents for the Pb(II) removal from real polluted water samples.

Based on the aforementioned information, the current work was performed with the following goals: (i) preparation of *H. Salicornicum* derived adsorbents in the raw form (HS) and the mercerized form (HSN); (ii) characterization of the prepared adsorbents using FTIR, Boehm titration, SEM, and TGA & DTG; (iii) batch adsorption experiments using Pb(II) ions as potential pollutants; (iv) investigation of the experimental variables affecting adsorption of Pb(II) onto the *H. Salicornicum*-derived adsorbents; (v) illustrating isotherm, kinetic, and thermodynamic parameters; (vi) studying statistical error validity on isotherm and kinetic models; (vii) elucidation of the Pb(II) adsorption mechanism onto the HS and HSN adsorbents.

## Experimental

### Materials and solutions

All the chemicals and reagents were sourced from Merck in analytical grade. A stock solution of 1000 (mg/L) Pb(II) was made by dissolving 1.598 g of the lead nitrate in deionized H_2_O and adding a few drops of conc. HNO_3_ to inhibit hydrolysis. The solution pH was adjusted using 0.1 M of HCl and/or NaOH. A 1000 mg/L solution of 4-(2-Pyridylazo) resorcinol (PAR) was prepared by dissolving 1 g of PAR in 1 L of deionized water.

### Preparation of *H. Salicorncium (HS)* plant-derived adsorbents

#### Preparation of Raw HS plant-derived adsorbent

The aerial parts of the HS plant (Fig. S1) were collected from naturally growing populations in Wadi Ash-Shaykh, Northeastern Desert, Egypt (28° 40’ 4.67” N 31° 3’ 58.47 E). The collected plant samples were cleaned from any impurities with de-ionized water and air-dried at room temperature (25 ± 3 °C) in shade conditions for 7 days. The dried plant parts were ground into fine powder for analytical use.

#### Preparation of NaOH modified *H. Salicorncium* (HSN) adsorbent

Alkali modification of HS was performed by immersing (30 g) of HS in approximately one liter of NaOH solution (0.5 M) for 24 h and shaking occasionally. After multiple decantations and filtration, the modified HSN adsorbent was washed with deionized H_2_O until the solutions reached pH 7.0. Then, the modified HSN adsorbent was dried in an oven at 105–110 °C until it became completely dry.

### Instrumentation and characterization

A HANNA pH meter (Hi 931401, Portugal) was used to measure the pH of the sample solutions. The adsorption studies, kinetics, and thermodynamic parameters were studied using a water bath shaker (NE5, Nickel-Electro Ltd., UK). UV-Vis spectrophotometer (JENWAY Co., Ltd., UK) is used for spectrophotometric determination of the remaining concentrations of Pb(II) solutions using PAR at λ_max_ = 520 nm.

The surface morphology of adsorbents was estimated by SEM using a JSE-T20 microscope (JEOL, Japan) with an acceleration voltage of 40 kV. FTIR spectra of the prepared adsorbents were recorded between (4000) and (400) cm^−1^ using a Shimadzu 5800 Fourier transform FT-IR spectrometer. The thermal stability of Sahara plants derived adsorbents was studied by using a Thermo-Gravimetric Analyzer (Shimadzu, Japan) under N_2_ (g) flow with a heating rate of 10 °C/min, between 25 and 800 °C. Using the traditional Boehm titration method, the surface chemistry and acid/base groups of the HS and HSN were determined^39^. About 0.1 g of the adsorbent samples were shaken with 0.1 N sodium hydroxide, sodium carbonate, sodium bicarbonate, and hydrochloric acid solutions then they were shaken for 48 h. After that, about 5 mL of each supernatant solution was titrated against (HCl and NaOH), using pH indicators, i.e., phenolphthalein and methyl red solutions depending on the originally used titrant. The surface pH of the adsorbents was determined as follows: about 0.1–0.5 g of the samples were shaken in 25 mL pre-boiled free CO_2_ de-ionized water for about 48 h and the pH of the supernatant was measured. Through applying the solid addition method, the adsorbents’ point of zero charge (pH_PZC_) was assessed. Determination of pH_PZC_ is done by shaking (0.1–0.15) g of the prepared samples with (0.01 M NaCl) solutions which were adjusted by 0.05 M HCl or 0.05 M NaOH between 2 and 12 for 48 h at 120 rpm at 25 °C, then the final pH was measured. The resulting pH was taken as the pH_pzc_. The final pH was plotted against ∆pH. At pH_PZC_, there is no change in solution pH that occurred during the equilibration period. Ash content is calculated as in Eq. (1) and is defined as follows: the measure of minerals presents as impurities in the samples^40^. Moisture content, Eq. (2), is a measure of the adsorbed water by samples stored under humid conditions. Some adsorbents may adsorb as much as 25–30% moisture and still appear dry^41^.

1 $${\text{Ash}}\;{\text{content}}\;\% =\frac{{{\text{weight}}~\left( {{\text{Final}}} \right)\left( {\text{g}} \right)}}{{{\text{weight}}\left( {{\text{Iintial}}} \right)~\left( {\text{g}} \right)}} \times 100$$

2 $${\text{Moisture}}\;{\text{content}}\;\% =\frac{{{\text{loss}}\;{\text{in}}\;{\text{weight}}~\left( {\text{g}} \right)}}{{{\text{initial}}\;{\text{weight}}~\left( {\text{g}} \right)}} \times 100~$$

### Procedures

#### Adsorption studies

The adsorption experiments of the Pb(II) were performed in 125 mL stoppered bottles that contained 25 mL of Pb(II) solution and an adsorbent dose (0.025 g). Then, these bottles were shaken at 150 rpm on a thermostated shaker at room temperature. After the equilibrium was reached, the solutions were centrifuged at 3000 rpm. The supernatant solution that includes the remains of the Pb(II). Various parameters were studied, such as contact time (10–350 min), temperature (25–60 °C), dose (0.005–0.08 g), pH (from 2.0 to 6.0), ionic strength, foreign ions and initial concentration of (5.0–350 mg/L). The Pb(II) removal percentage (R, (%)) and Pb(II) adsorption capacity (q_e_, (mg/g)) were estimated as mentioned in Eqs. (3) and (4), respectively.

3 $${q_e}=\frac{{\left( {{C_o} - {C_e}} \right)V}}{W}$$

4 $$R\% =\frac{{\left( {{C_o} - {C_e}} \right)}}{{{C_o}}} \times 100$$

Where, V (L), W (g), C_o_ (mg/L) and C_e_ (mg/L) are the Pb(II) solution volume, the weight of the adsorbent, the Pb(II) initial concentration, and equilibrium concentration, respectively.

#### Kinetics, isotherm and thermodynamics studies

*Effect of the initial concentration and isotherm adsorption studies* the equilibrium isotherm of Pb(II) was investigated by varying the Pb(II) initial concentration from 5.0 to 350 mg/l, which was agitated with 0.025 g of HS and HSN samples in 25 mL of solutions at room temperature for 24 h.

Adsorption isotherm experiments were performed at T of (303 ± 3 K) employing 0.01 g of adsorbent, Pb(II) concentrations (10.0–200 mg/L), shaking time (180 min), and pH (5.0). In the current work, the experimental data were analyzed using the Langmuir and Freundlich isotherms. The following Eqs. (5) and (6), respectively, provide their linear versions:

Langmuir isotherm model^11,42^:

5 $$\frac{{{c_e}}}{{{q_e}}}=\frac{1}{{b{q_m}}}+\frac{{{c_e}}}{{{q_m}}}$$

Freundlich isotherm model^43^:

6 $$\log {q_e}=\log {K_F}+\frac{1}{n}\log {C_e}$$

where q_m_ represents the system’s maximum adsorption uptake (mg/g), and q_e_ represents the adsorbed Pb(II) amount onto the surface of HS and HSN adsorbents (mg/g) at equilibrium. C_e_ (mg/L) is the Pb(II) concentration at equilibrium. b (L/mg), K_F_ ((mg/g)⋅(L/mg)^1/n^), and n are Langmuir, Freundlich, adsorption affinity, and adsorption intensity constants, respectively.

*Effect of contact time and kinetic studies* The contact time effect is used to investigate the kinetics of the adsorption process. 0.025 g of Sahara plant-derived adsorbents was added to a series of bottles containing 25–50 mg/L of Pb(II) at constant pH (4.5–5). Then, the solutions were filtered and analyzed spectrophotometrically at different periods of time ranging from 10 to 350 min.

In the present study, the four kinetic models (pseudo-1st -order, pseudo-2nd -order, intra-particle diffusion (IPD), and Boyd’s models) were estimated in order to investigate the rate, mechanism, and adsorption rate-controlling step^9,11^.

The following Eqs. (7–10), respectively, provide their non-linear versions:

Pseudo-1st-order equation:

7 $$\log ({q_{e1}} - {q_t})=\log {q_e} - \frac{{{k_1}}}{{2.303}}t$$

Pseudo-2nd-order equation:

8 $$\frac{t}{{{q_t}}}=\frac{1}{{{k_{2~}}q_{{e2}}^{2}}}+\frac{1}{{{q_{e2}}}}~t$$

Weber–Morris IPD equation:

9 $${q_t}={k_{int~}}{t^{0.5}}+c$$

Boyd’s model equation:

10 $$F\left( t \right)=1 - \frac{6}{{{\pi ^2}}}\mathop \sum \limits_{{n=1~}}^{\infty } \frac{1}{{~~{n^2}}}{\text{~exp}}\left( { - {n^2}Bt} \right)$$

where q_e_ and q_t_ represent the adsorption capacity at equilibrium and contact time t (mg/g), the rate constants for the pseudo-1st and pseudo-2nd-order equations were k_1_ (1/min) and k_2_ (g/(mg/min)), respectively, whereas k_int_ was the rate constant for IPD (mg/(g/min^1/2^) and C represents the thickness of the boundary layer, *F* is the fractional of equilibrium at different times (t), and $B~\left( t \right)$ is the mathematical function of$~F$. *n* is an integer that defines the infinite series solution and $F~$is the equilibrium fractional attainment at time *t*.

*Statistical error validity on isotherm and kinetic models (The goodness of fit)* The best-fitting models cannot be found by applying only the correlation coefficient (R^2^) but can be estimated by using other forms of model validity evaluation^44^. The goodness of fit was assessed employing the following error functions: The chi-square statistic (χ2), the sum of squares error (SSE), and the mean square error (MSE) error, which are represented in Eqs. (11–13), respectively. They are investigated in similar adsorption studies^44–46^.

11 $${{\text{\varvec{\upchi}}}^2}=\mathop \sum \limits_{{i=1}}^{n} \frac{{{{\left( {{q_{e~i}}~exp - {q_{e~i}}{\text{cal}}} \right)}^2}}}{{{q_{e~i}}~cal}}$$

12 $$SSE=\mathop \sum \limits_{{i=1}}^{n} {\left( {{q_{e~i}}~exp - {q_{e~i}}{\text{cal}}} \right)^2}$$

13 $$MSE=\frac{1}{{{N_{exp}}}}\mathop \sum \limits_{{i=1}}^{n} {\left( {{q_{e~i}}~exp - {q_{e~i}}{\text{cal}}} \right)^2}~$$

Where *cal* and *exp* subscripts refer to theoretically calculated and experimental data, respectively. Meanwhile, n is the number of observations included.

*Effect of temperature and thermodynamics* The nature of Pb(II) adsorption on the investigated materials was estimated by studying the (ΔG^o^), (ΔH^o^), and (ΔS^o^) thermodynamic parameters, which are known as standard free energy, heat of enthalpy, and entropy of the adsorption process, respectively.

The values of ***∆*H**^**o**^ and ***∆*S**^**o**^ were assessed from the slope$\left( {\frac{{ - \Delta {{\text{H}}^0}}}{{\text{R}}}} \right)$ and the intercept $\left( {\frac{{\Delta {{\text{S}}^0}}}{{\text{R}}}} \right)$ of Van’t Hoff equation (Eq. (14)). While, ΔG^o^ was calculated using Eq. (15)^47^.

14 $$\ln {{\text{K}}_{\text{c}}}=\frac{{ - \Delta {{\text{H}}^{\text{o}}}}}{{{\text{RT}}}}+\frac{{\Delta {{\text{S}}^{\text{o}}}}}{{\text{R}}}$$

15 $${\text{\varvec{\Delta}}}{{\text{G}}^{\text{o}}}= - {\text{RTLn}}{{\text{K}}_{\text{C}}}$$

R: universal gas constant (8.314 × 10^− 3^ kJ/mol K). T: the temperature in (K), The spontaneous and feasibility nature of the adsorption process is determined by Thermodynamic variables. K_C_: thermodynamic equilibrium constant. ΔG^o^: the free energy. ΔH^o^: the heat of enthalpy. ΔS^o^: the adsorption entropy.

#### Sample analysis

Different environmental samples, such as distilled, ground, and tap water samples, as well as some food samples, were spiked with known amounts of Pb(II) and shaken with 0.025 g of adsorbents for 24 h, then they were filtered. Food samples were digested utilizing concentrated nitric acid at high temperatures. The obtained supernatant was then determined spectrophotometrically using PAR to determine the equilibrium concentration of Pb(II) ions.

#### Desorption study

The desorption investigation was performed to investigate the reusability and the desorption capacity of the prepared adsorbents. After the adsorption was completed, the adsorbents were gently washed with water to remove any unadsorbed Pb(II). Then, the samples were dried at (105–110) °C. A 0.025 g of (HS and HSN) was shaken with different pH solutions, which were adjusted from 1.5 to 12 with solutions (HCl and NaOH). The solutions were filtered and the desorption % of the adsorbate was calculated as follows:

16 $${\text{De-sorption}}\% =\frac{{amount~desorbed~~\left( {mg/l} \right)}}{{amount~adsorbed~\left( {mg/l} \right)}} \times 100$$

## Results and discussion

### Materials’ design and physicochemical properties of adsorbents

#### Alkali modification of adsorbents

One important variable to consider when applying adsorption is the activation of the adsorbents in order to increase their adsorption efficiency by increasing the surface area and adding functional groups^48^. The most popular method of activating the adsorbents is to use heat, vacuum, or superheated steam to extract the adsorbed gases. Other commonly utilized techniques are either to break down the adsorbent into smaller sizes or to roughen the adsorbent surface. Adsorbents can also be activated using chemicals. NaOH and KOH are widely used compounds in activating adsorbents. It was reported that activation utilizing NaOH enhances the adsorbents’ characteristics as it allows them to have a larger total pore volume and surface area in addition to smaller pore diameters^49^. Also, the activation by hydroxide compounds such as NaOH, KOH, and CaOH provides the –OH functional groups, which leads to better adsorption capabilities^50^. Herein, the alkali modification process of HS was carried out at a NaOH concentration of 0.5 M for 24 h. The adsorption efficiency of HS was increased after their treatment with NaOH. This may be returned to the impurities’ elimination which leads to increasing active functional groups like C–OH and C–O. In addition, the alkaline treatment causes increasing swelling capacity. Among a wide range of alkaline agents, NaOH is the most used one. This may be returned to its ability to disintegrate the lignocellulosic’ internal structure leading to the enhancement of material pore structure. The alkaline treatment of raw fibers with NaOH enables the hydroxyl group ionization to the alkoxide^51^. In conclusion, the treatment of waste biomass through the alkaline modification process has main advantages including the reduction of waste biomass hydrophobicity (results in easier removal of traces of organic solvents without the need for use at high temperatures), it can be obtained at ambient conditions (makes the process low-cost), and the increase of the superficial functional groups dissociation degree (results in an increase of the in the adsorption efficiency)^52^.

#### Ash and moisture content

Both ash and moisture contents are essential, as they can affect the samples’ adsorption properties. Ash is the inorganic, inert, amorphous, and unusable part presented in the activated sample that comes initially from the basic material. Moreover, the moisture content is the amount of physically bound water on the activated sample (HSN) under normal conditions. They can reduce the overall activity of the activated sample, i.e., the lower the ash and moisture value, the better the activated sample as an adsorbent. Not only can ash and moisture content affect the activated sample’s adsorption capacity, but also surface pH, point of zero charge, and the functional groups of the plant samples can affect it. All the investigated plants’ surface characterizations are present in Table 1.

**Table 1:** Physicochemical characters of the HS and HSN adsorbents.

Plant sample	Moisture content %	Ash content %	pH of supernatant	pH_pzc_	Bohem titration (mmol/g)			
					Carboxylic	Lactonic	Phenolic	Total basic
HS	9.26	0.175	5.87	6.17	0.8	0.034	1.966	1.85
HSN	7.79	4.08	6.6	6.61	0.896	0.304	1.7	2.25

As present in Table 1, the high moisture content (7.7 and 9.26%) in all HS and HSN gives an indication of having high surface area and plentiful active sites available on the surface of the prepared adsorbents and the presence of hydrophilic functional group on the hydrophobic composite surface. The higher ash content after modification may be due to the incorporation of sodium metal in plant morphology^53^.

#### Boehm’s Titration

Boehm’s titration was utilized for the acidic and basic groups investigation for the HS and HSN adsorbents. The traditional titration of the Boehm technique was used for the prepared adsorbents’ surface chemistry through acid and base groups’ determination. It is established on the surface functionalities neutralization upon their acid strength, as it is known that a functional group of a given pK_a_ can only be neutralized by a base having a higher value of pK_a_.

The data of Boehm’s titration of HS and HSN samples are recorded in Table 1. It was observed that the total surface basic sites are higher than the acidic sites. This confirms the basic nature of the HS and HSN surfaces, which agree with the results of pH_pzc_ and pH_sup_.

### Characterization

#### SEM

**Fig. 1:**
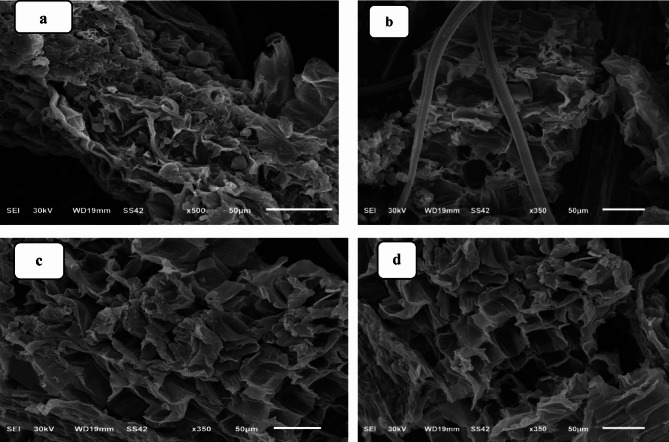
SEM images of (**a** and** b**) HS, and (**c** and** d**) HSN adsorbents.

SEM morphology explains how the activation affects the surface morphological properties of all samples. In the case of the raw *H. Sallicornicum* (HS) plant as shown in Fig. 1a and b and comparing the images after modification with sodium hydroxide in Fig. 1c and d, there is a noticeable change in the surface morphology, after modification compared to the native materials of the plants. From Fig. 1, it can be observed that all the adsorbents have rough textures with heterogeneous surfaces and a variety of randomly distributed pore sizes.

#### FTIR spectra

**Fig. 2:**
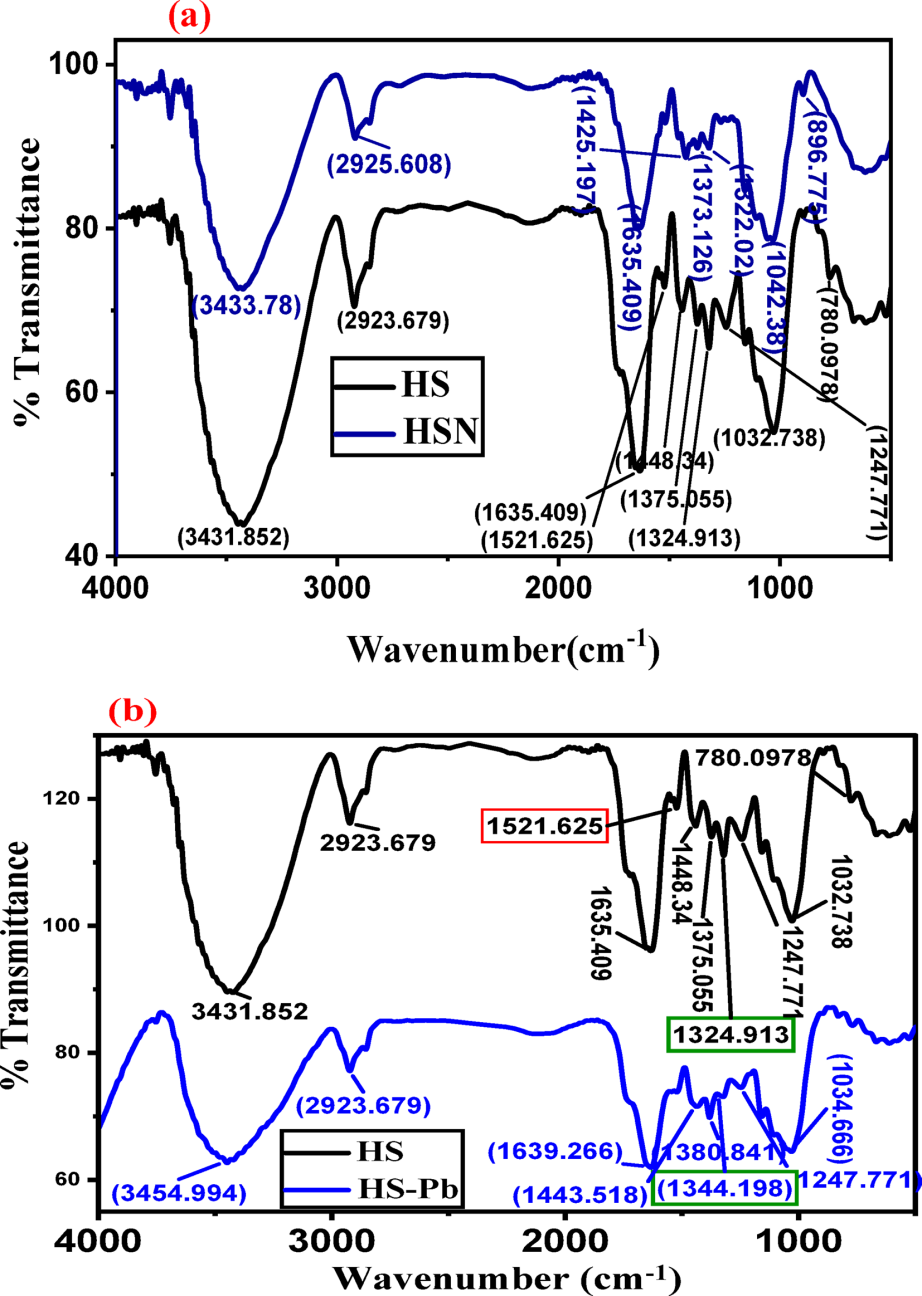

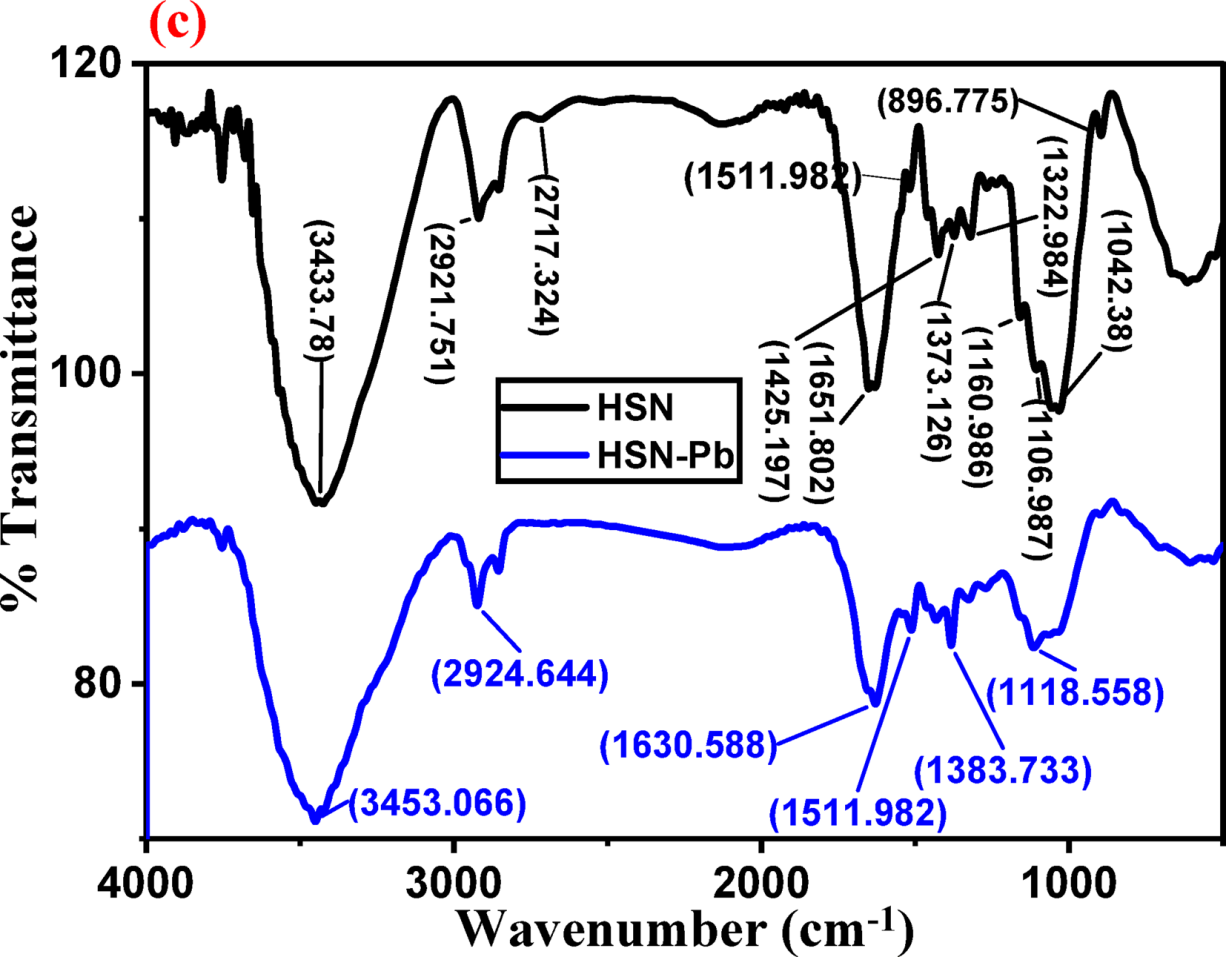
FTIR spectra of **a** HS compared with HSN, **b** HS compared with HS-Pb, and **c** for HSN compared with HSN-Pb.

The importance of natural compounds in heavy metals removal, stems from having various functional groups like –OH, –COOH, –C=O, and amine groups. The FTIR spectra of HS and HSN before and after adsorption of Pb(II) are shown in Fig. 2a–c. The FTIR of HS and HSN before adsorption of Pb(II) are shown in Fig. 2a. The absorption bands at 1635 cm^−1^ in the case of the HS sample attributed to the (C=O) stretching vibration of carboxylate ion (–COO^−^) groups in the organic compounds^54^. The peak between 1511 and 1521 cm^−1^ in both samples is attributed to (C=C) of aromatic rings of the lignin esters, which is a stable band in all lignin structure material^55^. The two observed peaks around 1373–1375 and 1425–1448 cm^−1^ represent (C–H) methyl groups of lignin that are associated with hemicellulose. In the case of the HSN sample, the peak at 1651 cm^−1^ represents C=O stretching vibration in quinones^56^ present in cellulosic plants. For HS, the peak at (1324) cm^−1^ represents methylene (–CH_2_–) and hydroxyls (O–H) groups of aromatic groups. Absorption bands at 1247 cm^−1^ in the case of HS and 1160 cm^−1^ in the case of HSN are attributed to C–O stretching vibration due to hemicellulose and aryl groups in lignin^57,58^, respectively. The peak at 1032 cm^−1^ in HS is attributed to C–O group stretching vibration in phenol, acids, ethers, and esters in plants. In the case of the HS and HSN samples, the small peaks at 1032 and 1106 cm^−1^ are due to the C–O contribution in glycosidic linkage and the C–O stretching in phenols, alcohols, acids, ethers, and esters, respectively^43,59,60^.

The FTIR spectra of HS and HSN adsorbents after Pb(II) adsorption are shown in Fig. 2b and c. There are various changes in the absorption bands, where the peaks of some bands are shifted, such as (3431 and 3433) cm^−1^ in both samples are shifted to (3453 and 3454) cm^−1^; in the case of the HS sample, the peaks at 1635, 1448, 1375, and 1324 cm^−1^ are shifted into 1639, 1443, 1380, and 1344, respectively. Noticeable changes can be observed in the FTIR spectra of HS and HSN before and after adsorption.

The availability of functional groups (from FTIR spectra) and the more roughness and HSN surface porosity in Fig. 1 allowed the enhanced Pb(II) adsorption efficiency from aqueous solutions.

#### Surface pH and pH_PZC_

**Fig. 3:**
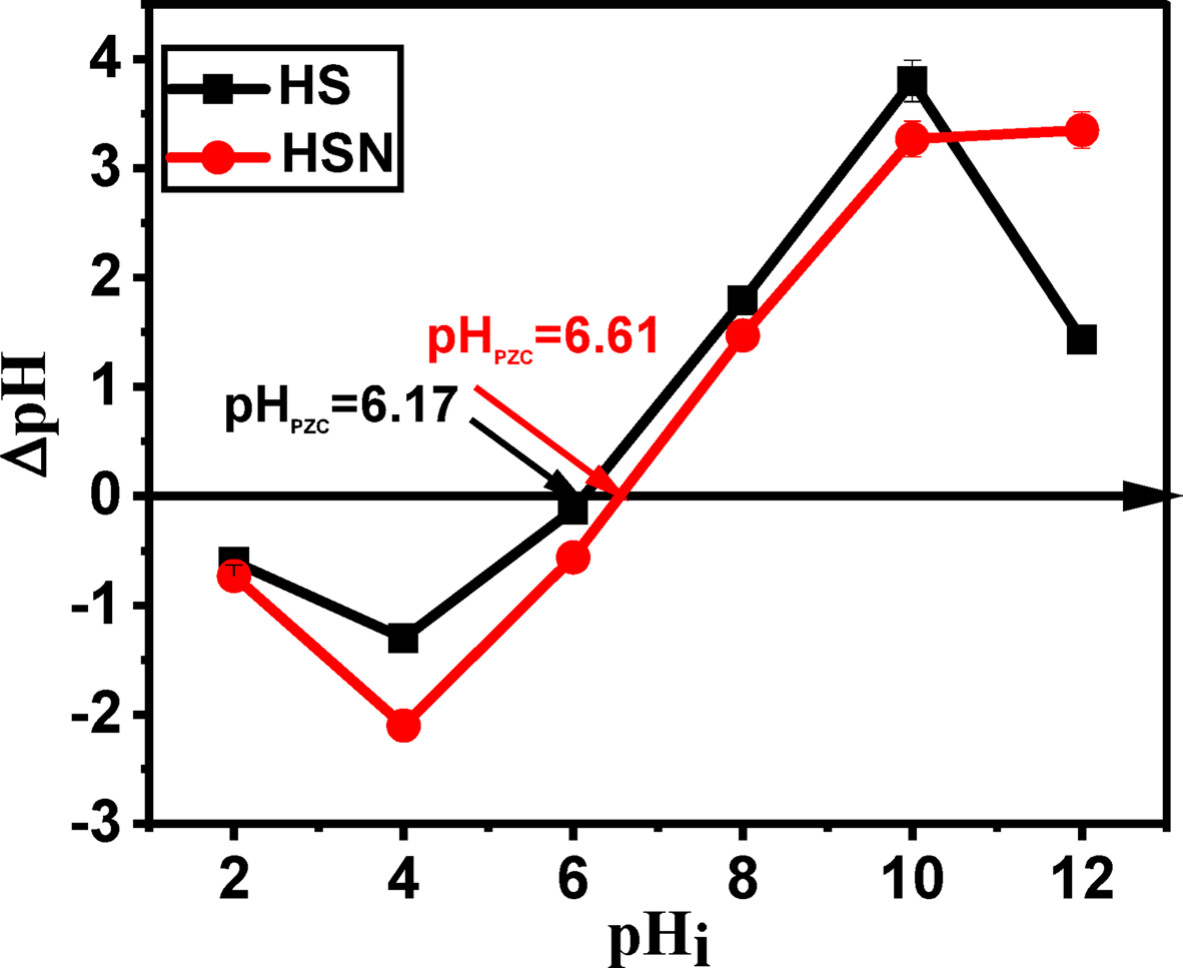
pH_PZC_ for (filled square) HS and (filled circle) HSN adsorbents.

As the surface is positively charged, the surface sites are protonated at a pH higher than pH_PZC_. Meanwhile, at a value lower than pH_PZC_ is negatively charged. It was determined by the solid addition method^61^. From Table 1, the pH of supernatant in the case of plant samples was 6 and 6.6 for HS and HSN, respectively. After activation, there is a slight increase in pH, which may be attributed to the basic functional group on the sample surface during the treatment. Bronsted acidic groups of the adsorbent surface in solution tend to donate their protons to water molecules, and the surface becomes negatively charged. On the other hand, Lewis bases took protons from the solution, hence becoming positively charged. It is known that most of the oxygen-containing functionalities behave as Bronsted acids, donating protons to the aqueous media and so being responsible for pH < 7 of adsorbents^62^. It is known that the basic properties arise from two types of contributions: the adsorption of protons by the aromatic basal planes of the plants and by surface complexes. The plots to determine pH_PZC_ are shown in Fig. 3, and the estimated values of pH_PZC_ are shown in Table 1.

#### Thermal analysis

**Fig. 4:**
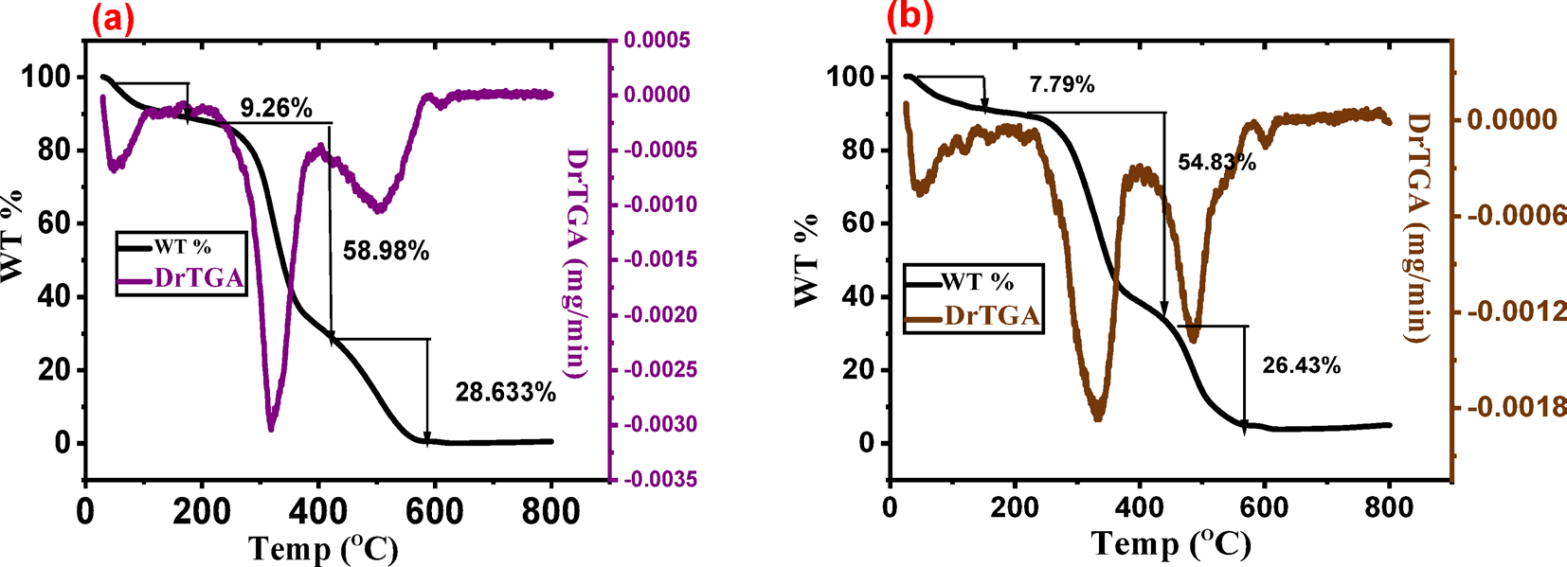
Thermal analysis of, **a** HS, and **b** HSN adsorbents.

Figure 4 shows the TGA of the Sahara plant-derived adsorbents. From the TGA profile, the initial step shows a weight loss of (9.26 and 7.79%) in the temperature range of 33–177 °C, which may be attributed to the release of moisture and volatile matter present in such plants in the following order (HS and HSN). These are Sahara plants and sure they contain different cellulosic materials, so the second step shows a gradual weight loss of (58.98 and 54.83%) at the temperature range from 217 to 436 °C which may be associated mainly with the decomposition of hemicellulose and other low molecular weight materials of them in the same order. The third step involved a small loss in weight at a temperature above 400 °C, which may be attributed to the decomposition of cellulose, and continued up to 700 °C due to the decomposition of lignin with weight loss of (28.63 and 26.43%), respectively.

### Adsorption studies

#### Effect of pH

**Fig. 5:**
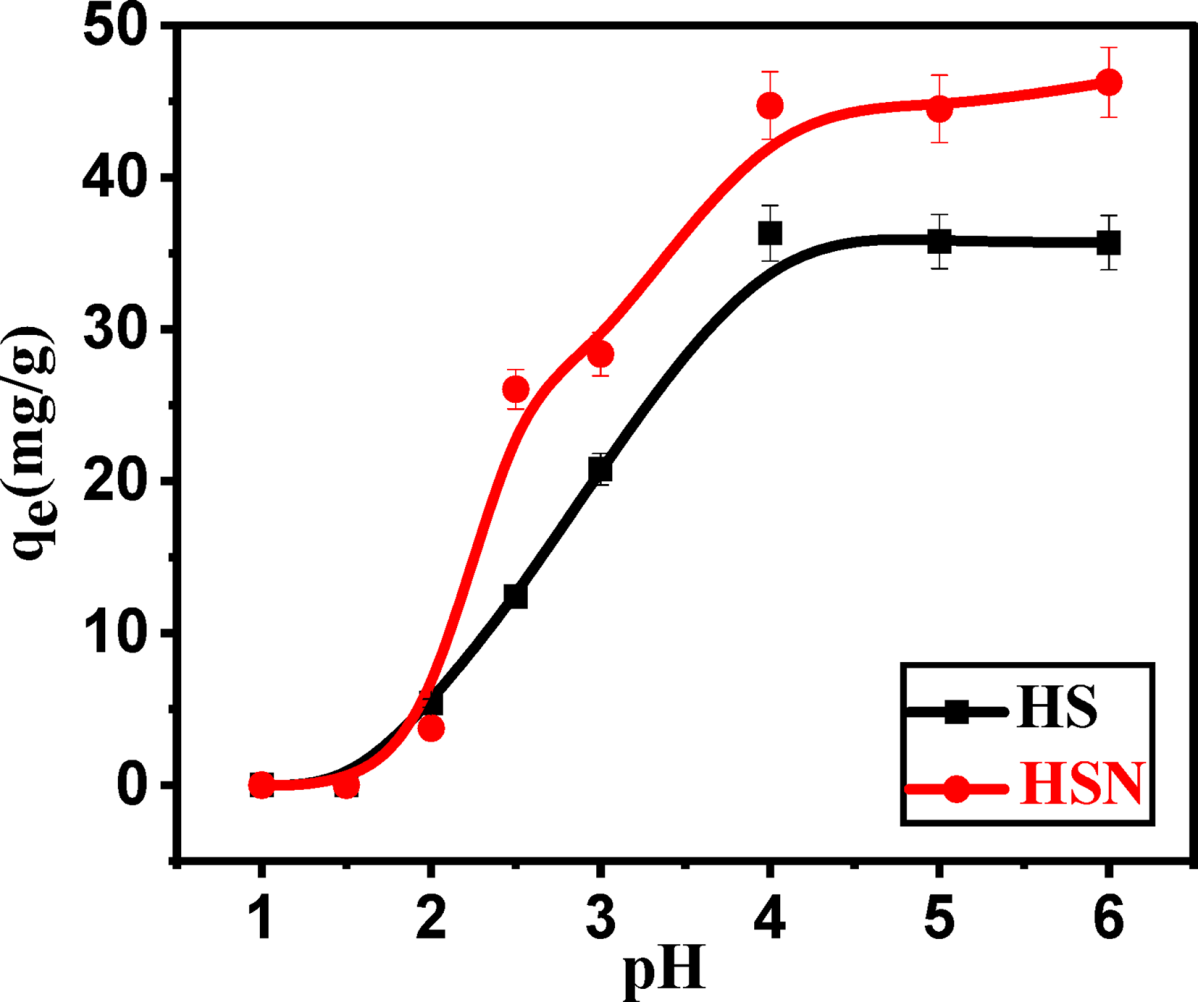
Effect of pH on adsorption capacity of Pb(II) on () HS and () HSN adsorbents.

The pH of the aqueous solution is one of the essential variables influencing the adsorption processes, as it controls the adsorbent’s surface charge and ionization level in addition to the adsorbate speciation. Pb(II) adsorption employing HS and HSN adsorbents was examined in the pH ranges between (2 and 6.5) to investigate how the pH solution affects the Pb(II) removal. Removal of Pb(II) ions above pH (6.5) is not favored since, in these conditions, Pb(II) is precipitated as Pb(OH)_2_ species^63^. Figure 5 illustrates that the adsorption capacity of the Pb(II) is significantly influenced by the solution pH. It was observed that the HS and HSN adsorption capacity for Pb(II) increases with solution pH increase, and a maximum value at pH equal to 5 was achieved. These results suggest that the adsorption of the Pb(II) attributed to the HS and HSN adsorbents’ surface properties. As pH values increase, the quantity of negative charges on the adsorbents increases, leading to the complete ionization of active sites. Consequently, the electrostatic attraction between the active sites and the Pb(II) intensifies. Accordingly, increasing pH makes more negatively charged surfaces available, facilitating more Pb(II) removal. In reverse, the Pb(II) adsorption efficiency diminishes at low pH values due to an increase in hydrogen ions on the HS and HSN surfaces and the occurrence of electrostatic repulsion between the HS and HSN surfaces and Pb(II) in solution. This phenomenon may be linked to lyophobic behavior between the adsorbent (HS and HSN) and adsorbate (Pb(II)), altering their forces^64^. At pH values higher than 6.0, Pb(II) ions are precipitated as their hydroxides, which reduces the adsorption rate and, in turn, the percentage of metal ions removed. It was observed that the maximum adsorption capacity was achieved at pH (4.5–5.0).

#### Effect of adsorbent dose

**Fig. 6:**
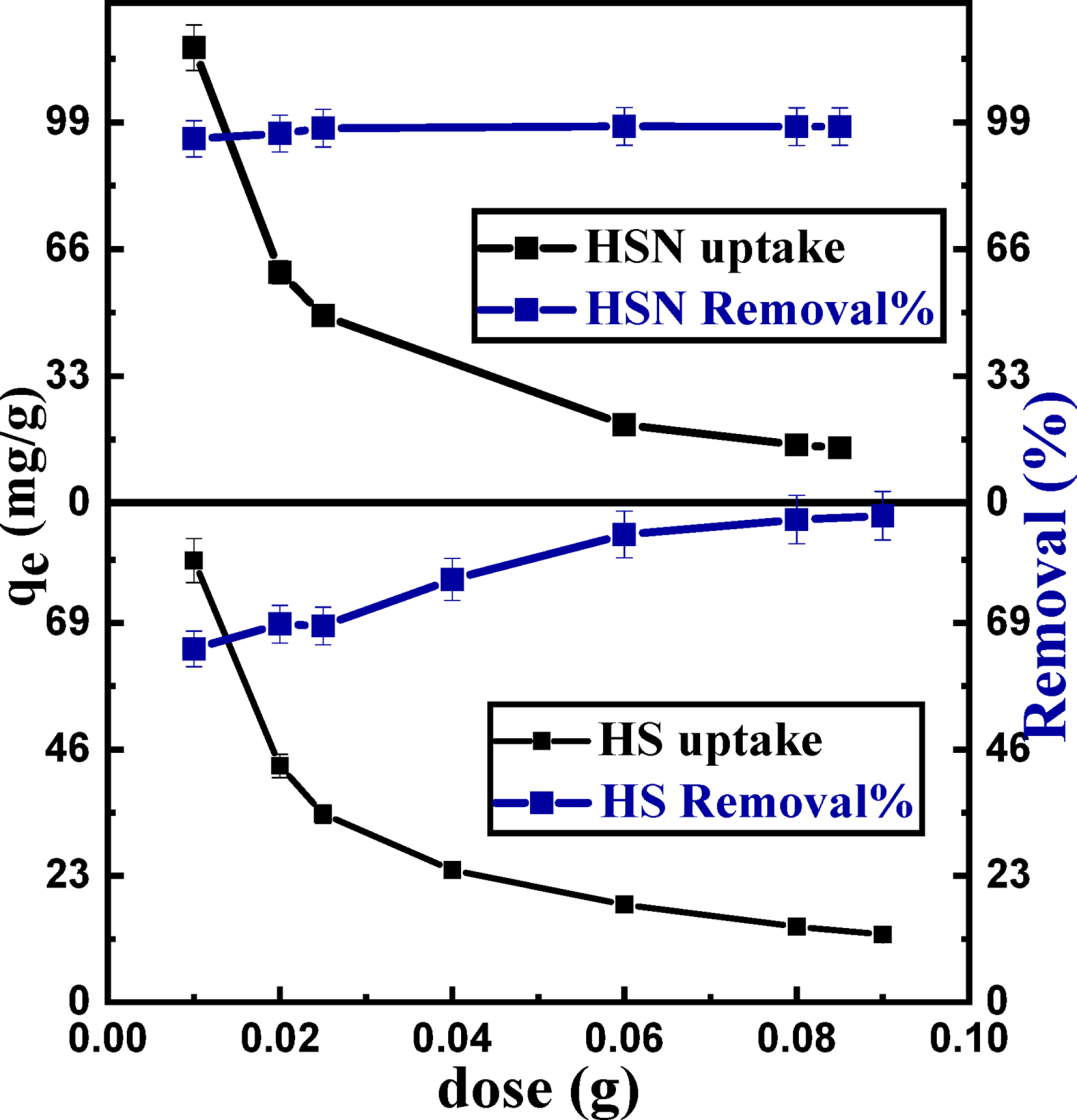
Effect of adsorbent dosage on Pb(II) ions removal.

The adsorbent dose effect was investigated by varying the HS and HSN samples dose from 0.02 to 0.1 g at 50 mg/L Pb(II) solution. As illustrated in Fig. 6, the Pb(II) adsorption increases with increasing the HS and HSN adsorbent dose. As shown in Fig. 6, the removal percentage of Pb(II) ions increases from 64.14% to 94.02% at 0.01 g of HS and HSN adsorbents, respectively, to reach 88.38% and 97.58% at a dose of 0.08 g. Shortly with HS and HSN adsorbent dose increases, the available active sites increase, which leads to an elevation in Pb(II) ions sorption capacity. Here, the adsorption capacity (mg/g) showed the highest values of 80.37 and 118.42 mg/g at a dose of 0.01 g for HS and HSN, respectively.

#### Effect of initial concentration of Pb(II)

**Fig. 7:**
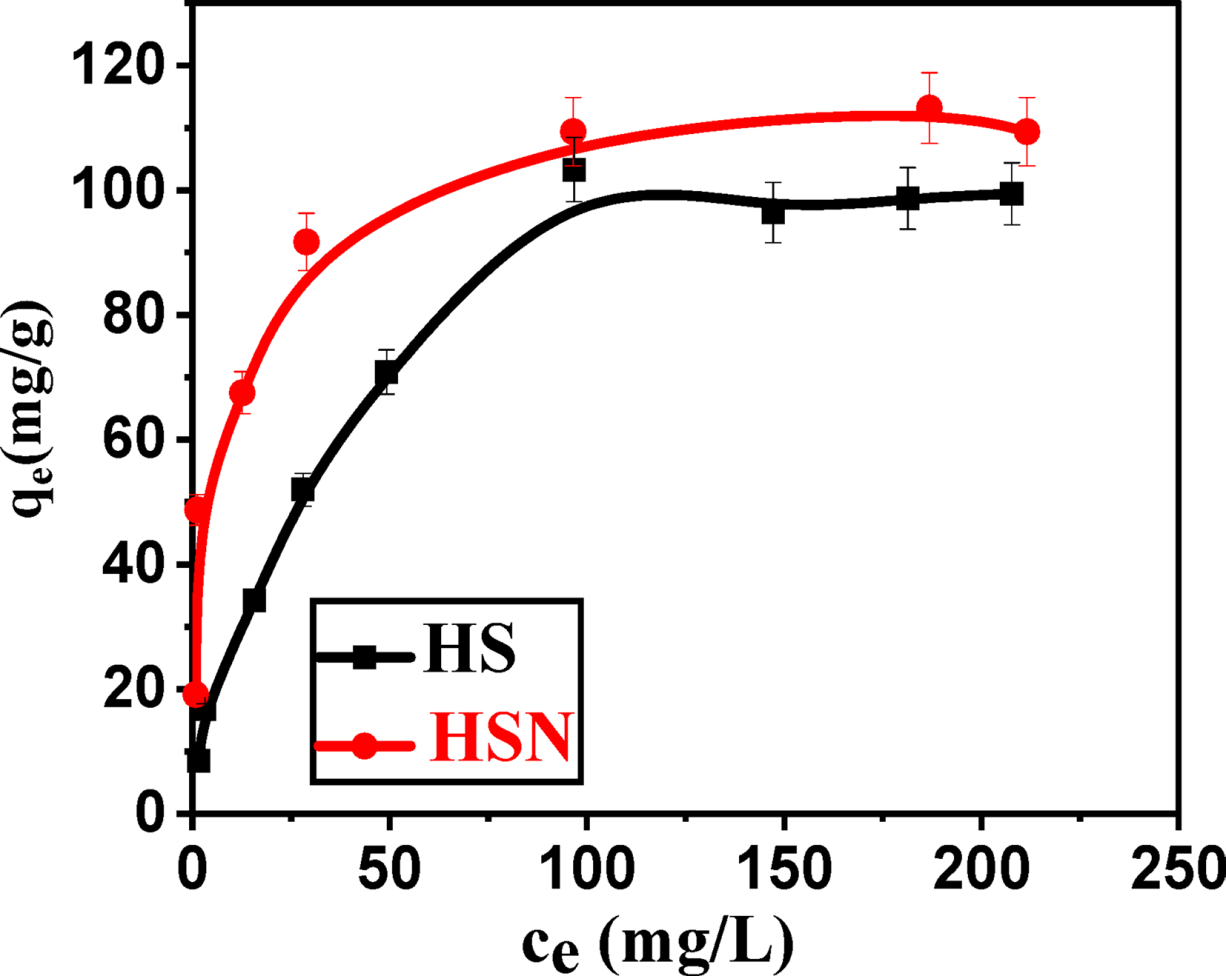
Effect of initial Pb(II) concentration on adsorption capacity of () HS and () HSN adsorbents.

As observed in Fig. 7, the adsorption capacity for Pb(II) increased from 8.46 to 103.3 and 19.18 to 109.4 mg/g for HS and HSN, respectively, with Pb(II) initial concentration increasing from 5.0 to 100 mg/L. This is attributed to the available adsorption vacant sites at lower initial Pb(II) concentrations, where at high initial concentrations Pb(II), there is a decrease in both adsorption capacity and removal percentage of Pb(II) ions due to the saturation of the available active sites of adsorbents^42^. Moreover, with the increase of Pb(II) initial concentration from 150 to 350 mg/L, the HS and HSN adsorption capacities tend to stabilize. As the initial concentrations of Pb(II) ions become more than available active sites of adsorbents, no more adsorption of Pb(II) ions can be achieved, which results in stability of adsorption capacity in Pb(II) ions removal percentage.

#### Adsorption isotherm studies

**Fig. 8:**
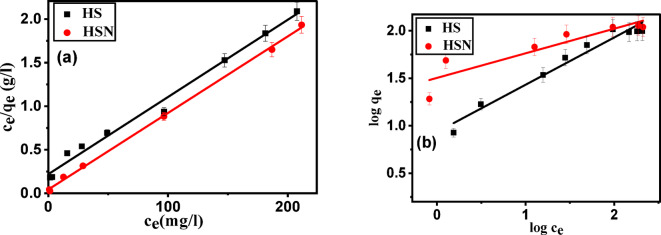
Adsorption isotherm model for Pb(II) ions (**a**) Langmuir and (**b**) Freundlich.

In order to comprehend the adsorption process, the adsorption isotherm is utilized as a fundamental tool. The type of surface phase, monolayer or multilayer, is an important factor that adsorption isotherm analytical forms depend on. The intricacy of these models is associated with the structural and energetic heterogeneity of sorbent material surfaces, which is an element of a large number of adsorbents employed either in experiments or in industry^65^. The linear fitted curves are shown in Fig. 8, and the data for each isotherm model of Pb(II) adsorption on the Sahara plants derived (HS and HSN) adsorbents are reported in Table 2.

The Langmuir isotherm model, which suggests a monolayer uptake on the homogeneous surface of the adsorbent^9,11^, is the best-fitted model for the Pb(II) adsorption onto the prepared Sahara plant-derived (HS and HSN) adsorbents, according to the highest values of R^2^ ≥ **0.995** and the lower error functions. In this investigation, the Langmuir isotherm was used to estimate the monolayer adsorption capacities (q_m_) of Pb(II), which were found to be 112.86, 113.76 mg/g for HS and HSN, respectively. In addition, the characteristics of Langmuir isotherm R_L_, the separation factor, and a dimensionless constant are defined as follows:

$${R_L}=\frac{1}{{1+b{C_O}}}$$

When the value of R_L_ is between 0 and 1, the adsorption process is favorable. It is unfavorable when R_L_ is more than 1, irreversible when R_L_ is zero, and linear when R_L_ is 1. By analyzing the generated data, it can be shown that the R_L_ values are within 0 and 1, suggesting highly favorable adsorption of Pb(II) onto the prepared Sahara plant-derived adsorbents^66^.

**Table 2:** Langmuir and Freundlich isotherm constants for Pb(II) ions adsorption.

Model	Parameter	Adsorbent	
		HS	HSN
Langmuir	q_m_ (mg/g)	112.86	113.76
	b (L/mg)	0.040	0.201
	R^2^	0.987	0.998
	R_L_	0.0819	0.016
	χ^2^	288.106	139.218
	SSE	32515.674	15837.510
	MSE	3612.852	2262.501
Freundlich	K_f_ ((mg/g)⋅(L/mg)^1/n^)	8.59	31.87
	1/n	0.497	0.258
	R^2^	0.960	0.800
	χ^2^	338.112	139.2
	SSE	41259.487	15837.5
	MSE	4584.387	2262.5

#### Effect of contact time and adsorption kinetic studies

**Fig. 9:**
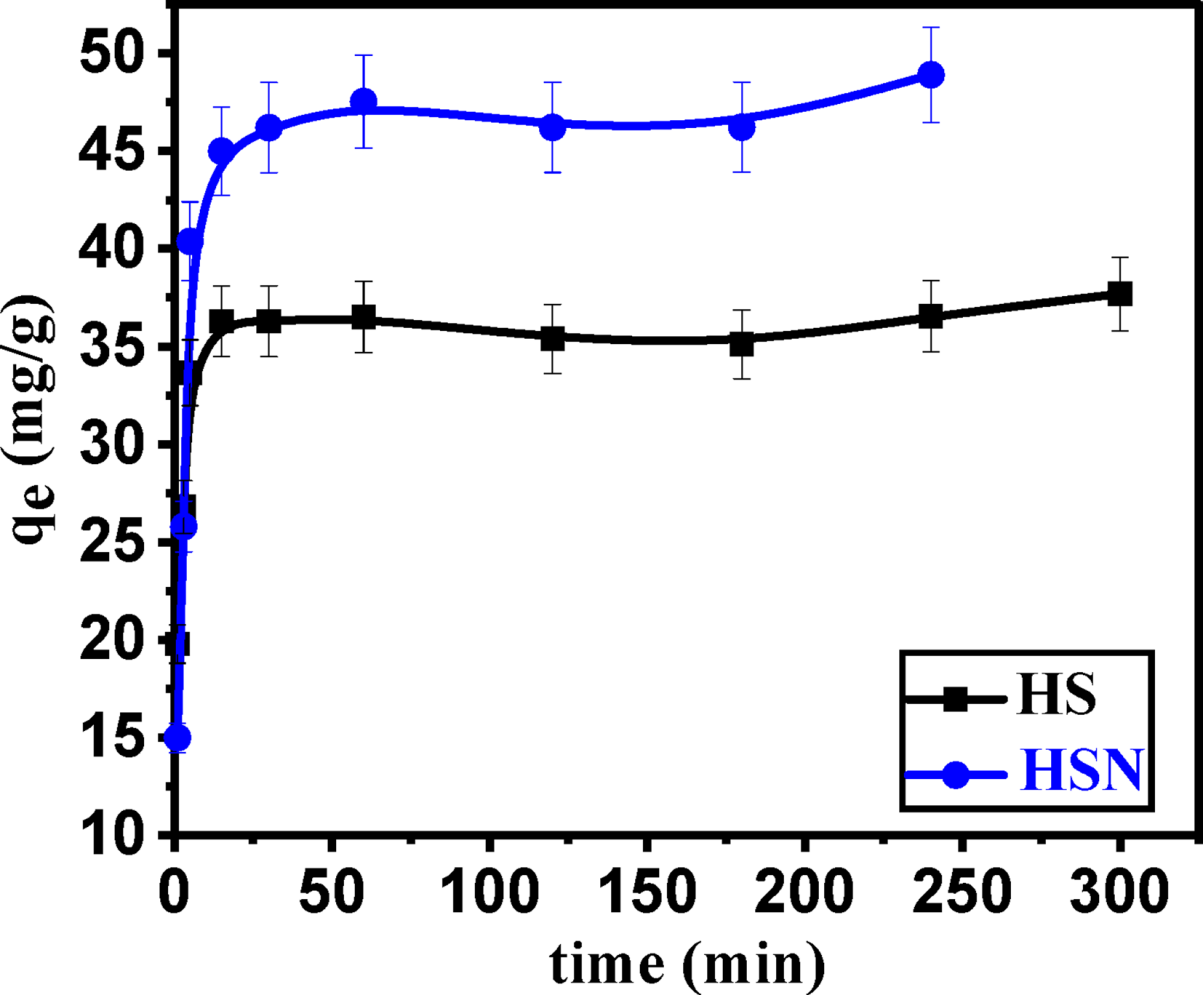
Effect of contact time on adsorption of Pb(II) ions of () HS and () HSN adsorbents.

The effect of contact time on the removal and adsorption capacity of Pb(II) solution is shown in Fig. 9. The removal % of Pb(II) increases rapidly at the first 30 min because of the availability of unoccupied active sites of the adsorbent surface. After that, the removal % slowly increases till it becomes constant after 60 min when equilibrium was established at which the vacant active sites were occupied with Pb(II) molecules^67^. 2 h of contact time is chosen as the adsorption time for the experimental test to ensure that equilibrium is reached.

**Fig. 10:**
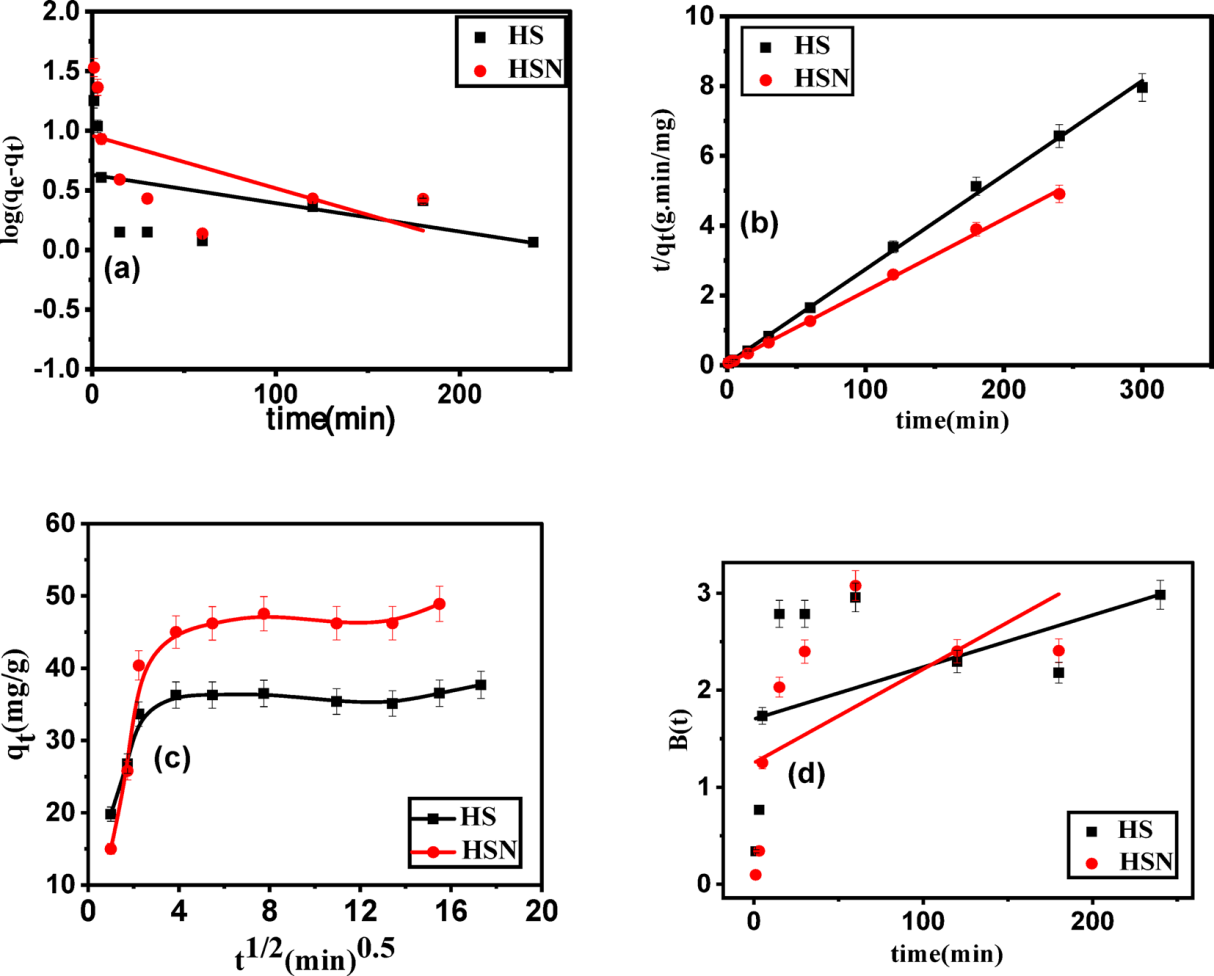
Kinetic models for Pb(II) adsorption: (**a**) pseudo-1st-order, (**b**) pseudo-2nd-order, (**c**) intra particle diffusion, and (**d**) Boyed’s model on the HS and HSN Sahara plants derived adsorbents samples.

The various kinetic parameters employed in this investigation are represented in Fig. 10a–d while Table 3 enlists the values of these parameters. When compared to the pseudo-1st-order parameter, the correlation coefficient value (R^2^) for the pseudo-2nd-order parameter was highest, as shown in (Table 3). Based on the highest correlation coefficient R^2^ (**0.998**–**0.999**), the pseudo-1st -order kinetic model seems to be the most suitable model for explaining the adsorption of Pb(II) onto Sahara plants derived adsorbents. Besides, the error functions’ values of the pseudo-2nd -order that are shown in Table 3 are significantly lower than those of the pseudo-1st -order, implying that the adsorption of Pb(II) onto HS and HSN adsorbents fits the pseudo-2nd-order kinetic model in a chemosorption manner^68^.

The multi-linearity of the adsorbate adsorption process was depicted in Fig. 10c, suggesting that two or more stages were involved in the adsorption process. Adsorbate external diffusion onto the adsorbent was responsible for the first linear step, while delayed intra-particle adsorbate diffusion was responsible for the second linear step. Establishing equilibrium was demonstrated in the third step. In the initial and final stages of adsorption, the variation of mass transfer causes the deviation from the origin. A process like external diffusion, film diffusion, or surface adsorption may also be involved in the adsorption process, as evidenced by the existence of multi-linearity and the thickness of the boundary layer. This suggests that IPD was not the sole rate-controlling step in the adsorption process. Moreover, Boyd’s graph in Fig. 10d didn’t go through the origin, indicating that the rate-limiting adsorption process for Pb(II) adsorption on HS and HSN is film diffusion.

**Table 3:** Kinetic parameters for the adsorption of Pb(II) onto HS and HSN Sahara plants derived adsorbents samples.

	HS	HSN
Kinetic parameter		
q_eexp_ (mg/g)	37.7	48.9
Pseudo-1st -order kinetic equation		
q_e1_(mg/g)	4.27	9.103
k_1_ (1/min)	5.48 × 10^− 3^	0.01
R^2^	0.125	0.233
χ^2^	10315.76	4710.2
SSE	44048.29	42862.84
MSE	4894.25	6123.3
PseudoSecond-2nd -order kinetic equation		
q_e2_ (mg/g)	37.02	48.23
k_2_ [g/(mg min)]	0.0155	9.90 × 10^− 3^
R^2^	0.998	0.998
χ^2^	492.066	307.06
SSE	18216.3	14,809
MSE	2024.032	2115.623
Intraparticle diffusion equation		
k_int_ [mg/(g min^1/2^)]	0.594	1.508
c (mg/g)	28.69	29.75
R^2^	0.321	0.394
Boyd equation		
Intercept	1.702	1.251
R^2^	0.125	0.239

#### Effect of temperature and thermodynamic studies

**Fig. 11:**
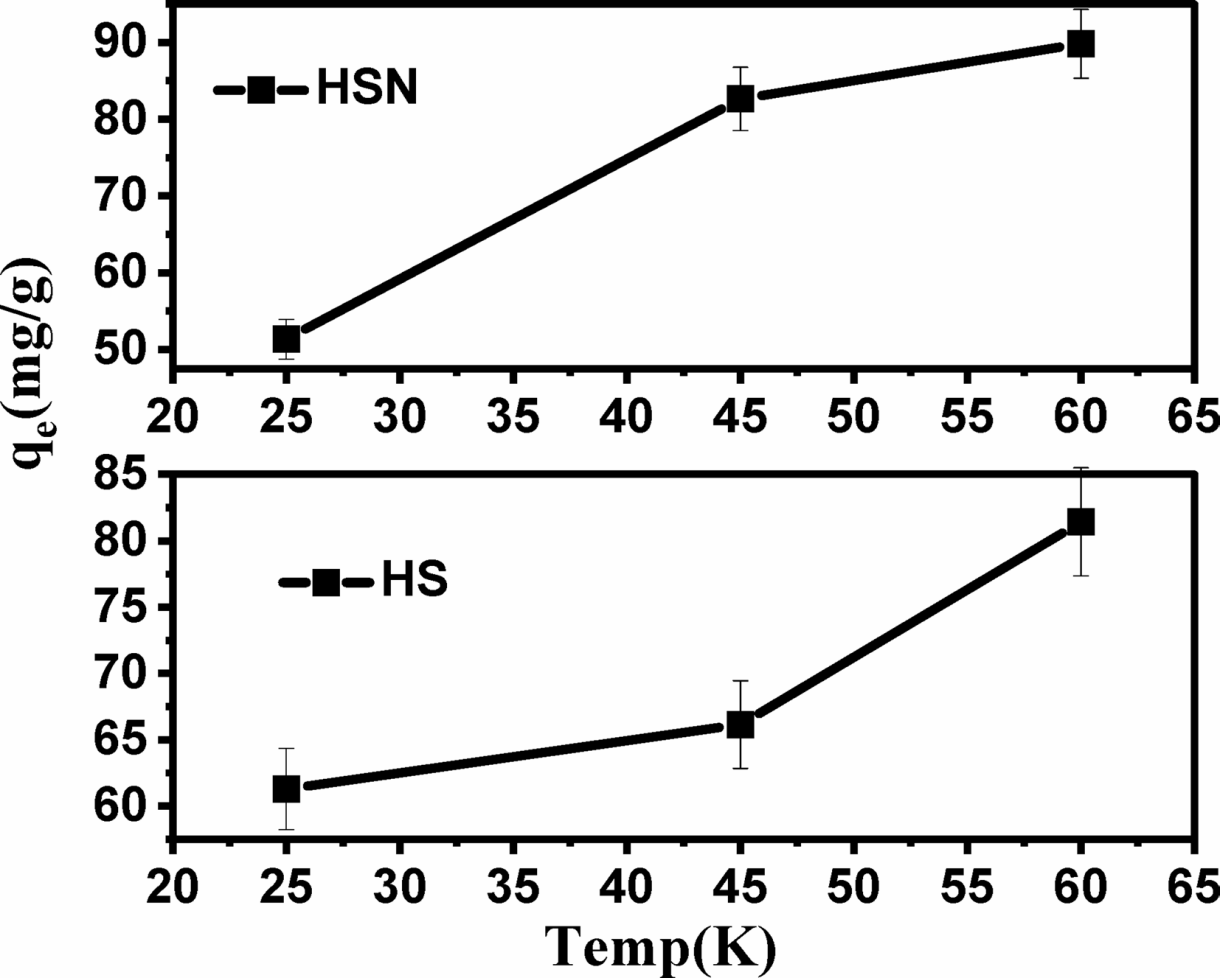
Effect of temperature on the adsorption capacity of Pb(II) on HS and HSN Sahara plant derived adsorbents.

Figure 11 presents the temperature influence on Pb(II) adsorption onto HS and HSN. It can be estimated that with temperature elevating, the HS and HSN adsorption efficiency increases, indicating that the adsorption process requires energy to occur and thus suggesting that adsorption is favored at higher temperatures^69,70^.

**Fig. 12:**
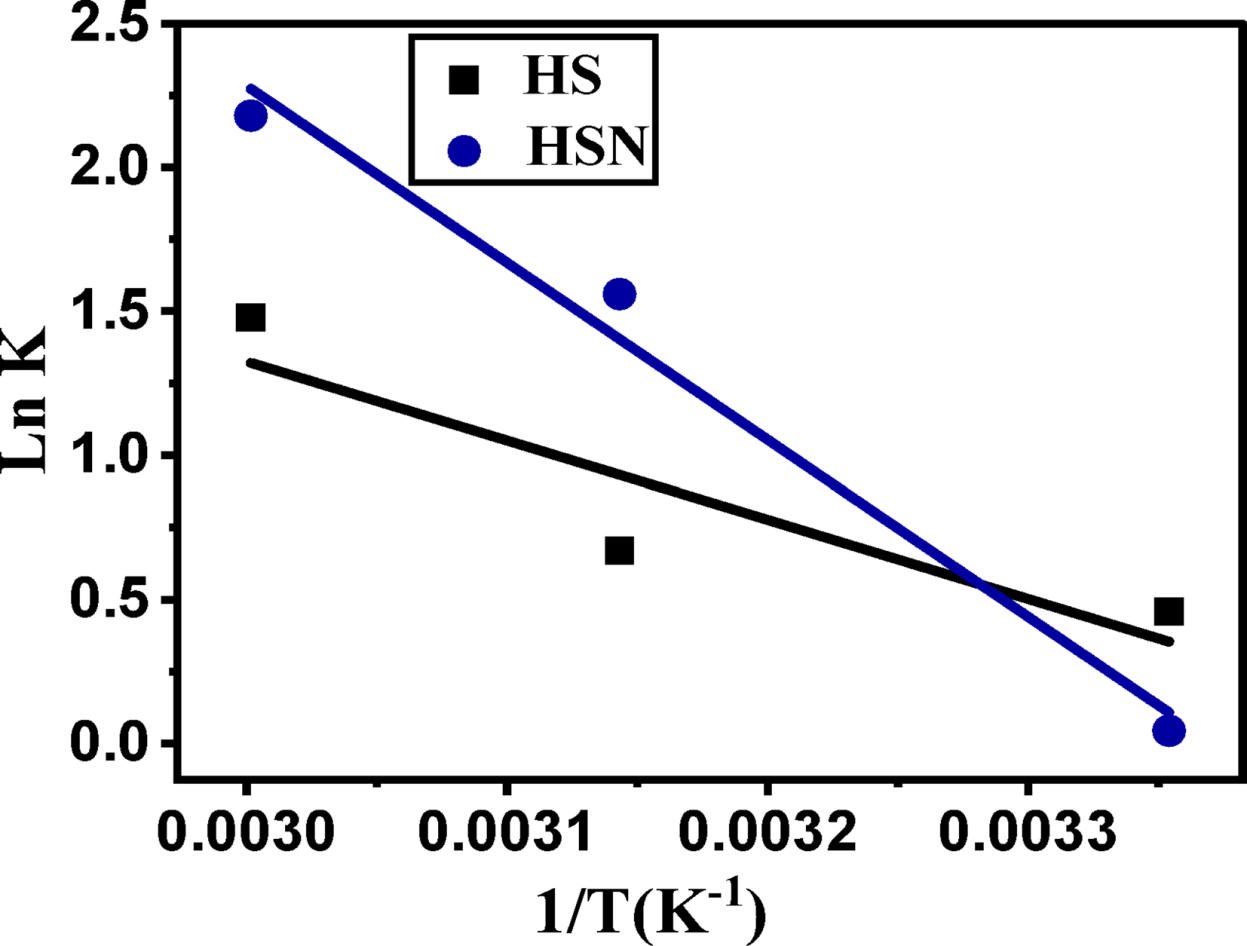
Van’t Hoff isotherm for adsorption of Pb(II) on HS and HSN adsorbents.

The data of ΔG^o^, ∆H^o^, and ∆S^o^ are tabulated in Table 4. From Table 4; Fig. 12, the negative ΔG^o^ value for HS and HSN samples indicates the thermodynamically feasible and spontaneous nature of the Pb(II) adsorption. The investigated adsorbents (HS and HSN) have positive ∆H^o^ values, indicating the endothermic nature of the adsorption process^71^. The positive ∆S^o^ value observed in the samples HS and HSN demonstrates an increase in randomness at the adsorbent-adsorbent interface during high-affinity adsorption of these activated patterns for Pb(II) with some structural changes in the adsorbent-adsorbent system for Pb(II) adsorption.

**Table 4:** Thermodynamic parameters for the adsorption of Pb(II) on HS and HSN adsorbents.

Adsorbent	K_c_			ΔG^0^ (kJ/mol)			ΔH^0^ (kJ/mol)	ΔS^0^ (J/molK)
	298.15 K	318.15 K	333.15 K	298.15 K	318.15 K	333.15 K		
HS	1.582	1.954	4.384	-1.137	-1.772	-4.094	22.82	79.50
HSN	1.045	4.760	8.841	-0.109	-4.127	-6.036	51.09	172.26

#### Effect of ionic strength

This effect was investigated at 20 mg/L of (II) on HS and HSN adsorbents and varying the NaCl concentration between (0.001–0.15) M at normal pH. The results obtained revealed that the metal removal increases with increasing NaCl concentration up to 0.05 M. By increasing NaCl solution concentration above 0.05 M, the Pb(II) removal decreases. The competition between Pb(II) and Na ions for the sorption available sites may be the main reason for the Pb(II) removal decrease at salt concentration > 0.05.

#### Effect of foreign ions

Various foreign ions’ influence on the Pb(II) removal (R, %) was investigated by utilizing Sahara-derived adsorbents. The results (Table 5) displayed that Pb(II) can be removed successfully using Sahara-derived adsorbents in the presence of different cations and anions species, such as F^−^, C_2_O_4_^2−^, CH_3_COO^−^, Ca^2+^, KH^4+^, and Mg^2+^.

**Table 5:** Effect of foreign ions on the Pb(II) elimination utilizing HS and HSN Sahara plants derived adsorbents.

	Removal %	
Plant samples	HS	HSN
Foreign ions 100 µg/ mL each		
Acetate^−^	91.9	90.95
Cl^−^	89.45	90
F^−^	93.3	87.1
Po_4_^3−^	95.2	95.5
NH_4_^+^	92.35	92.35
Na^+^	91.8	89.05
Ba^2+^	89.15	93.15
Mg^2+^	84.55	84.6

#### Desorption studies

Regeneration of HSN adsorbents is a source of an economical method for reusing them several times. Regeneration is based on the fact that this adsorbent is a stable material that is resistant to acidic and basic media and can withstand temperature changes. Also, due to increased environmental awareness and costs associated with the disposal of contaminated bio-residues, processes that can be reused or regenerated such adsorbents in situ have become more attractive. There are several advantages to regeneration. It reduces the environmental impact of the disposal of the spent adsorbents and reserves the landfills.

**Fig. 13:**
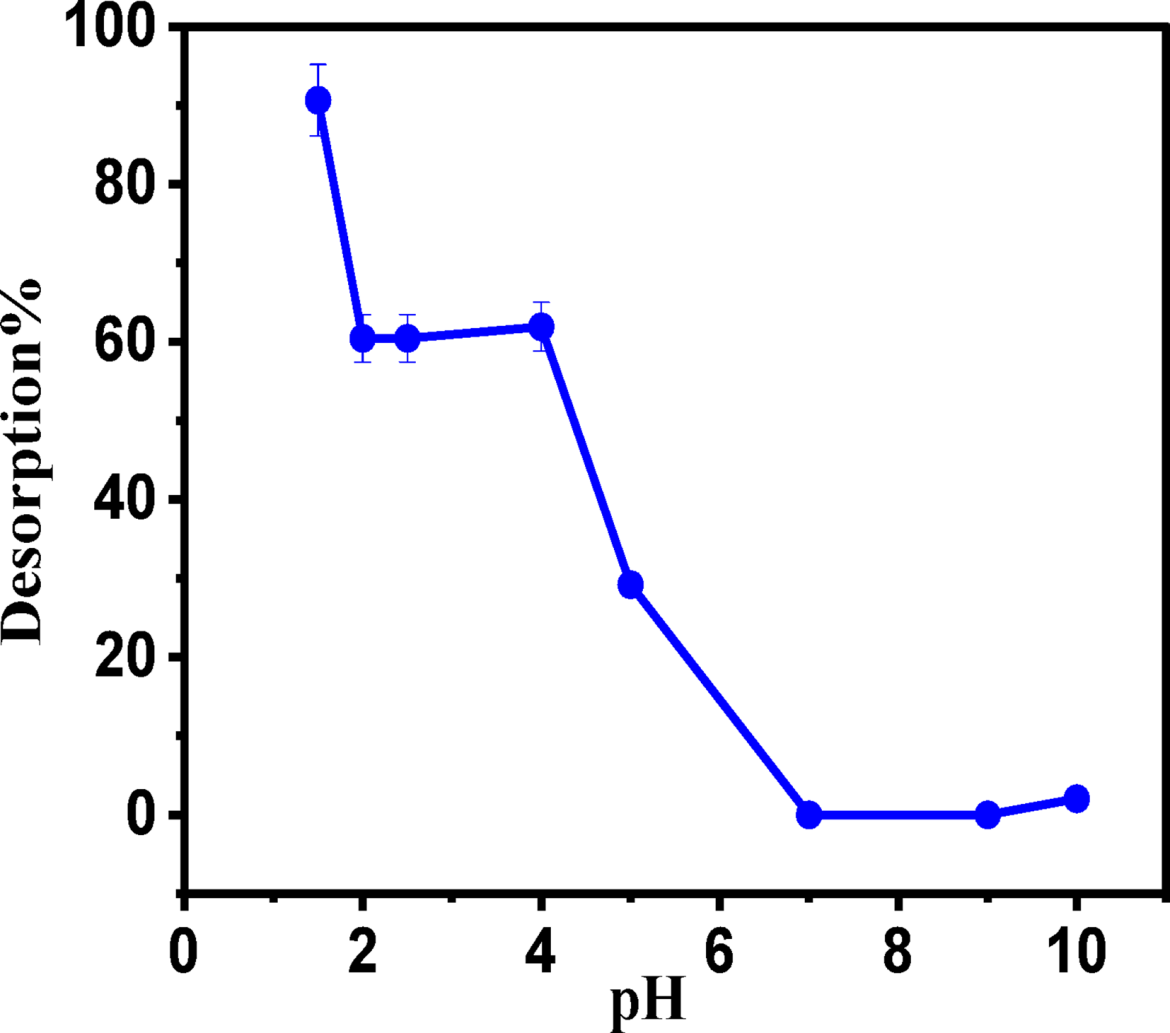
Desorption of Pb(II) from HSN by adjusting the pH values of the solution using HCl and NaOH.

In order to find if the prepared adsorbents can be reutilized or not, desorption studies were executed by changing the pH solution ranging from (1.5 up to 10) using HCl and NaOH. The desorption effect is shown in Fig. 13. The desorption efficiency decreased as the pH value of the solution increased. Desorption efficiency reaches 90.66% at (pH = 1.5) and decreases to reach 2.05% at (pH = 10). The above results show that the Pb(II) adsorbed by HSN biosorbent can be easily desorbed and can be employed repeatedly in heavy-metal water purification. Furthermore, the regeneration process also indicates that ion exchange is one of the main adsorption techniques.

#### Applications

The applicability of the prepared HS and HSN Sahara plant-derived adsorbents for Pb(II) removal from different environmental samples, such as (distilled, tap, and ground) water samples and digested food samples was investigated. Various Pb(II) concentrations were spiked in PVC flasks containing 25 mL of the mentioned environmental samples, and 25 mg of the biosorbent was added. The adsorption process was performed as previously mentioned. The filtrate, which contains the remaining Pb(II) ions, was measured spectrophotometrically utilizing the PAR ligand. Table 6 shows that the Pb(II) recoveries are satisfactory and that the HS and HSN adsorbents could be efficiently employed with high accuracy and precision to eliminate and determine Pb(II) ions in real samples.

**Table 6:** Pb(II) ions recovery from different environmental samples utilizing prepared HS and HSN Sahara plant derived adsorbents (*n* = 3).

Sample	Pb(II) added (mg/L)	(*R*, %)	
		HS	HSN
De-ionized water	0	–	–
	5	89.4	96
	10	84.62	95.9
	15	82.3	94.5
Tap water	0	–	–
	5	92.66	–
	10	51.88	–
	15	19.8	
Ground water	0	–	–
	5	83.72	–
	10	96.39	
	15	34.26	–
Flour	20	–	81.8
Rice	20	–	80.99
Herbal extract	20.1	–	83.09

#### Performance of the prepared on HS and HSN Sahara plants derived adsorbents

According to the literature, several materials have been investigated for their capability to remove Pb(II). In this study, we employed *H. salicornicum* in two forms, raw (HS) and mercerized (HSN), as adsorbent for the removal of Pb(II). A comparison between the investigated adsorbents (HS and HSN) and other existing adsorbents reveals several key advantages, like environmental sustainability, cost efficiency, high adsorption capacity, and availability. The HS is an agro-waste material, making it an environmentally friendly option for adsorption. Using agricultural waste not only decreases trash disposal difficulties but also supports ecologically friendly heavy metals’ treatment procedures. Cost efficiency is a key advantage for the current investigation because HS is less expensive than typical adsorbents; it is a feasible choice for industrial applications, especially in developing countries with tight budgets. Moreover, Preliminary results indicate that HS and HSN have comparable or even superior adsorption capacity for Pb(II) compared to other adsorbents like coconut coir and pokeweed (Table 7). This can be assigned to the HS biosorbent porous structure and the presence of several functional groups that facilitate the Pb(II) binding. In addition, HS is readily available in numerous regions, which can improve its relevance in different geographical contexts, providing a practical solution for heavy metal removal in wastewater treatment. A comparative investigation of the maximum adsorption capacity (q_e_) of Pb(II) utilizing various low-cost plant-derived adsorbents is presented in Table 7. These adsorbents are widely available and cheaper than synthetic resins. The calculated q_e_ demonstrates that HS and HSN exhibit noteworthy potential for effectively removing Pb(II) from aqueous solutions. This outcome underscores the environmentally friendly and efficient nature of HS as a biosorbent for addressing Pb(II) contamination in wastewater.

**Table 7:** The maximum sorption capacity of HS and HSN Sahara plants derived adsorbents for Pb(II) compared with previously published adsorbents.

Biosorbent	q_e_ (mg/g)	Biosorbent type	References
Pokeweed (untreated)	13.19	Plant	^72^
Pokeweed (treated with nitric acid)	14.51	Plant	^72^
Durian tree sawdust	20.37	Plant	^73^
Coconut coir	37.04	Plant	^73^
Oil palm empty fruit bunch	37.59	Plant	^73^
Olive tree pruning (untreated)	27.05	Plant	^74^
HS	100.68	Plant	Present study
HSN	113.33		

#### Plausible mechanism of adsorption of Pb(II) onto HS and HSN adsorbents

**Fig. 14:**
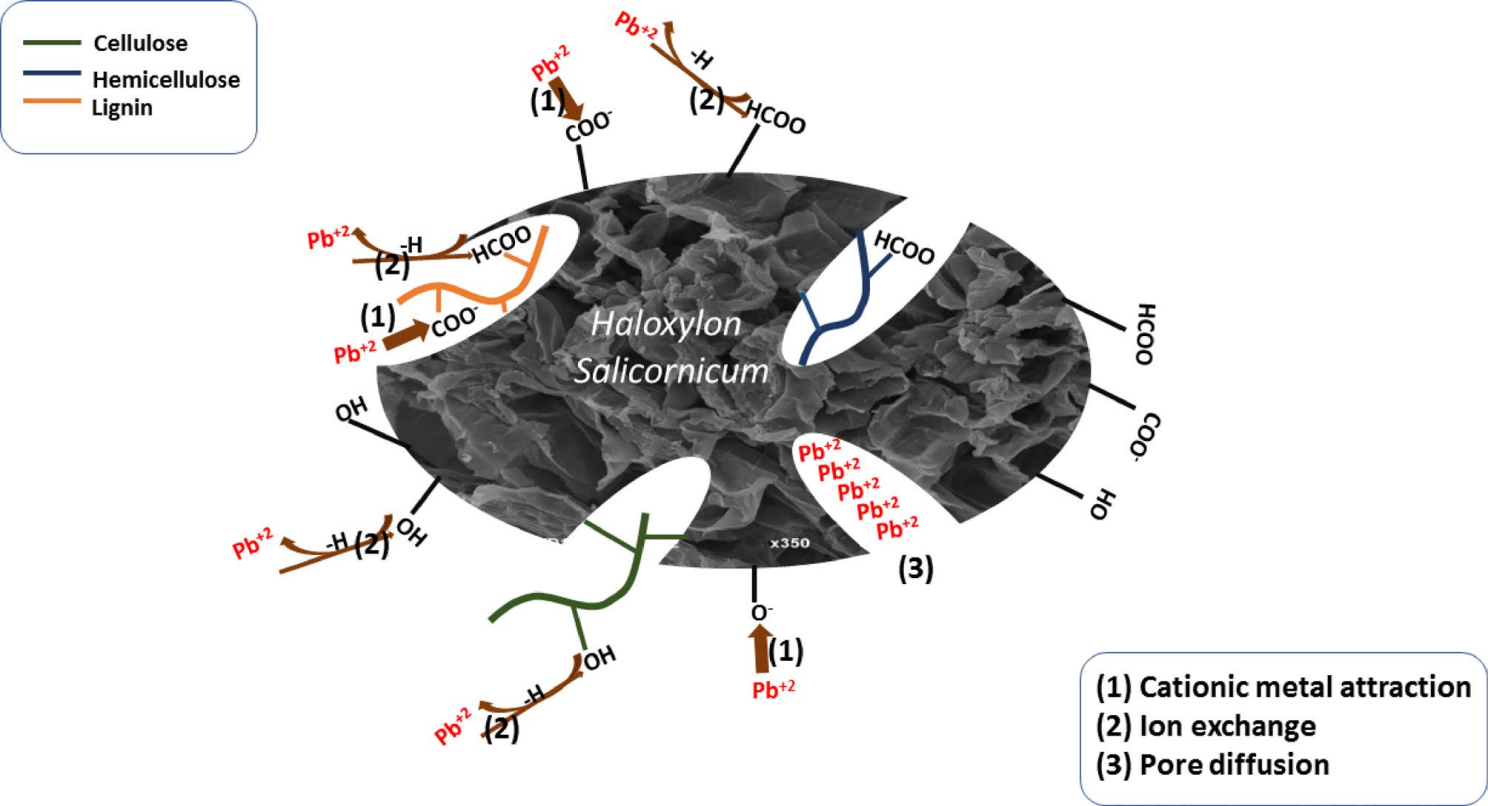
Plausible mechanism of sorption of Pb onto *H. salicornicum* Sahara plant.

The comprehensive elucidation of the Pb(II) adsorption mechanism onto HS and HSN unfolds through a synergistic strategy amalgamating experimental analysis and theoretical calculations. The illustrated representation of this intricate mechanism is presented in Fig. 14. The adsorption of Pb(II) onto HS and HSN adsorbents depends on several factors: the isotherm, kinetics, pH influence, and FTIR results. The main adsorption mechanisms of Pb(II) onto HS and HSN adsorbents include pore diffusion. In addition to the ion exchange, both HS and HSN surfaces are rich with various acidic groups, like carboxyl and phenolic hydroxyl, which can form complexes with Pb(II). Moreover, the electrostatic interaction, where positive charges on Pb(II) interact harmoniously with the negative ones on the investigated adsorbents (HS and HSN). Electrostatic adsorption depends on the formation of ionic bonds at a pH value higher than the pH_PZC_ of HS and HSN^75,76^. So, this thorough understanding of the adsorption mechanism provides invaluable insights, not only enriching our fundamental knowledge but also making provision for the optimization and customization of adsorption processes aimed at remediating lead-contaminated waters. These insights, illustrated in Fig. 14, shed light on the intricate mechanism driving the adsorption of Pb(II) onto HS and HSN adsorbents, enabling the development of more efficient and long-lasting remediation strategies.

## Conclusion

The study explores the potential of *H. salicornicum * Sahara plant derived adsorbents, in two forms raw (HS) and mercerized (HSN), as highly effective adsorbents for the removal of Pb(II) from aqueous solutions. The HS reveals remarkable promise because of its accessibility, cost-effectiveness, and superior adsorption capacity. The as-prepared adsorbents (HS and HSN) were characterized by surface area, Boehm’s titration method, FTIR, SEM, and TGA. Several essential parameters were investigated in order to understand the adsorption process fully, including oscillating time, initial Pb(II) concentration, HS and HSN adsorbent dose, initial pH of the Pb(II) solution, temperature, and salt ionic strength. The results indicate that the HS and HSN adsorbents were successfully utilized for the Pb(II) removal from numerous environmental samples. Furthermore, the optimum conditions for maximum adsorption capability are evaluated at a pH of 4.5 at 60 min oscillating time. The adsorption equilibrium investigations revealed that the Langmuir model fits well with adsorption capacities of 100.68 and 113.33 mg.g^− 1^ for HS and HSN, respectively. Adsorption kinetic studies indicated that the PSO model can best fit the data. Also, IPD is not the only rate-limiting step, suggesting the involvement of other processes influencing the adsorption rate of HS and HSN adsorbents. **χ**^**2**^, SSE, and MSE error functions were investigated and showed that PSO has lower values than PFO and Langmuir has lower values than Freundlich. Thermodynamic studies revealed the endothermic nature of adsorption of Pb(II) in the case of HS and HSN adsorbents. The adsorption-desorption investigation outcomes showed that the HS and HSN adsorbents have very good reusability. Finally, this study highlighted novel Egyptian Sahara plant *H. salicornicum* derived adsorbents which are rarely published in the water treatment process for Pb(II) removal via adsorption techniques.

## Electronic supplementary material

Below is the link to the electronic supplementary material.

Supplementary Material 1

## Data Availability

All data generated or analysed during this study are included in this published article.
