# Supplementary material for: Novel mercerized Haloxylon salicornicum Sahara plants derived adsorbents for efficient removal of lead(II) from wastewater
Source: Sci Rep. 2025 May 31;15:19136. doi: 10.1038/s41598-025-03791-1 (PMC12126575; doi:10.1038/s41598-025-03791-1)
Supplement: Supplementary file 1 — Supplementary Material 1 [file 41598_2025_3791_MOESM1_ESM.docx]

## **Novel mercerized *Haloxylon salicornicum* Sahara plants derived biosorbents for efficient removal of lead(II) from wastewater**

Magda A Akl^1^, Asmaa Serage^1^, Aya G Mostafa^1^ and Yasser Al-Amier^2^

^1^Department of Chemistry, Faculty of Science, Mansoura University, Mansoura 35516, Egypt

^2^Botany Department, Faculty of Science, Mansoura University, Mansoura 35516, Egypt

*Corresponding author

Prof Dr. Magda A Akl

Professor of Analytical Chemistry, Department of Chemistry, Faculty of Science, Mansoura University, Mansoura 35516, Egypt

e. mail: magdaakl@yahoo.com


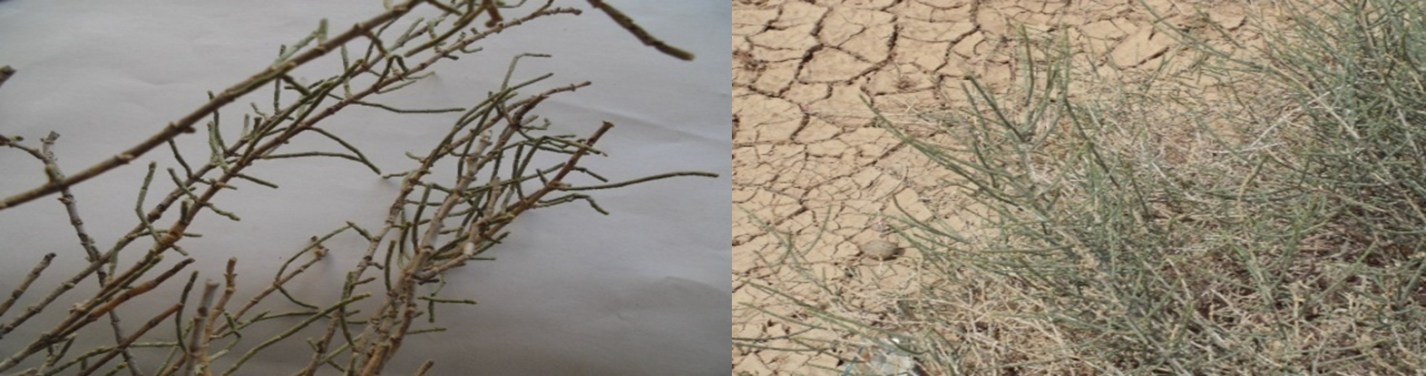
**Figure S1:** Haloxylon salicornicum
